# Efficacy, reliability, and patient satisfaction with Rigicon Infla10 X and Infla10 AX inflatable penile prostheses: a retrospective single-center analysis

**DOI:** 10.1093/sexmed/qfag058

**Published:** 2026-07-12

**Authors:** Bugra Cetin, Steven K Wilson, Britney L Atwater, Martin S Gross, Duygu Kirkik, Gokhan Yazici, Ozkan Onuk

**Affiliations:** Urology, Altinbas University Medicalpark Bahcelievler Hospital, 34160, Istanbul, Türkiye; Institute for Urologic Excellence, La Quinta, CA, USA; Section of Urology, Department of Surgery, Dartmouth Hitchcock Medical Center, Lebanon, NH 03756, United States; Section of Urology, Department of Surgery, Dartmouth Hitchcock Medical Center, Lebanon, NH 03756, United States; Rigicon Scientific Editor, Istanbul, Turkey; Department of Immunology, University of Health Sciences, Hamidiye Medicine Faculty, Istanbul, Turkey; Department of Urology, Biruni University Hospital, Istanbul, 34295, Türkiye; Department of Urology, Biruni University Hospital, Istanbul, 34295, Türkiye

**Keywords:** erectile dysfunction, penile prosthesis implantation, Rigicon Infla10, quality of life, patient satisfaction

## Abstract

**Introduction:**

Inflatable penile prosthesis implantation is a well-established treatment option for men with erectile dysfunction refractory to conservative therapies. However, patient-reported outcome data for the Rigicon Infla10 prosthesis remain limited. This study aimed to evaluate 12-month patient-reported outcomes following primary implantation of Rigicon Infla10 X and Infla10 AX devices and to explore potential differences according to implant model and pump type.

**Methods:**

This retrospective single-center, single-surgeon cohort study included 275 patients who underwent primary Rigicon Infla10 X or Infla10 AX implantation between 2021 and 2024 and completed 12-month follow-up. Erectile function was assessed using the International Index of Erectile Function–Erectile Function domain (IIEF-EF), while postoperative satisfaction was evaluated using the Quality of Life and Sexuality with Penile Prosthesis (QoLSPP) questionnaire. Ease of inflation and deflation was assessed using a 10-point numeric rating scale. Subgroup analyses were performed according to implant model and pump type.

**Results:**

Mean preoperative IIEF-EF score increased from 6.09 ± 1.47 to 24.07 ± 1.78 at 12 months. The overall mean QoLSPP total score was 4.19 ± 0.16. QoLSPP scores did not differ significantly between the Infla10 X and Infla10 AX groups (4.20 ± 0.18 vs. 4.18 ± 0.16; *P* = .436). Patients using the Pulse pump reported significantly higher ease-of-inflation and ease-of-deflation scores compared with those using the earlier-generation pump (both *P* < .001), whereas QoLSPP total scores were similar between pump groups (*P* = .821). The overall complication rate was 5.5%, and device infection occurred in one patient (0.4%).

**Discussion:**

Primary Rigicon Infla10 implantation was associated with substantial improvement in erectile function and favorable patient-reported outcomes at 12 months. Although the Pulse pump was associated with improved ease-of-use ratings, these differences did not translate into higher overall satisfaction scores. Interpretation of pump-related comparisons should be cautious because pump type was era-dependent and not randomly assigned. Prospective studies with longer follow-up are warranted to confirm these findings and further evaluate device-related outcomes.

## Introduction

Erectile dysfunction (ED), previously known as impotence, refers to the consistent inability to attain or sustain a penile erection sufficient for successful sexual activity.^1^ In population-based data, ED is common among men with type 2 diabetes and is associated with multiple clinical correlates.^2^ Common etiologies include diabetes mellitus (DM), cardiovascular disease, radical pelvic surgery such as prostatectomy, and Peyronie’s disease.^3–7^

For many patients, medical management of ED provides effective symptom relief with minimal side effects.^8^^,^^9^ Although medical therapies for ED—including PDE5 inhibitors and intracavernosal injections—are effective for many patients, some either fail to respond or experience intolerable side effects, thus requiring surgical intervention. Rather than being viewed strictly as a last-resort option, penile prosthesis implantation is increasingly considered earlier in the ED treatment pathway, supported by high postoperative satisfaction and patient preference for earlier implantation.^10^^,^^11^ IPP represents a definitive treatment for refractory ED, offering durable restoration of erectile function and documented improvements in quality of life.^12^^,^^13^ Owing to their high efficacy and reliability, IPPs have become a widely utilized surgical solution for ED.^14^ IPP implantation has been associated with favorable device survival rates compared with other implantable surgical therapies.^15^

Recent advancements in IPP technology have focused on enhancing mechanical reliability, minimizing infection risks, and improving user ergonomics.^16^ The Rigicon Infla10 X (girth-expanding) and Rigicon Infla10 AX (length and girth-expanding) models, introduced in 2019, incorporate features designed to improve mechanical reliability, infection resistance, and user ergonomics.^17^ The Pulse pump is a newer-generation pump system introduced later in the product lifecycle, with design features intended to facilitate handling and usability.^18^ Despite emerging safety and outcome reports, there remains limited real-world evidence specifically evaluating patient-reported outcomes after Rigicon Infla10 implantation, and comparative data examining pump-related usability within this platform are scarce.^19^ In addition, currently available studies on Rigicon prostheses remain relatively limited in terms of detailed patient-reported usability assessments and device-handling experience, particularly regarding inflation and deflation ergonomics.

Accordingly, this retrospective single-surgeon study aimed to evaluate patient-reported outcomes following primary Rigicon Infla10 implantation using routinely documented 12-month follow-up data. Beyond overall satisfaction, this study specifically aimed to characterize patient-reported device usability and handling-related outcomes in routine clinical practice. The primary endpoint was overall postoperative satisfaction assessed by the QoLSPP total score. Secondary endpoints included changes in erectile function (IIEF-EF), pump inflation and deflation ease measured on a 1-10 numeric rating scale (NRS), and perioperative complication rates. Exploratory subgroup analyses were performed according to implant model (Infla10 X vs. Infla10 AX) and pump type (earlier-generation Rigicon pump vs. Pulse pump). Specifically, we asked: Does the Pulse pump improve patient-rated inflation and deflation ease compared with the earlier-generation Rigicon pump? Does improved pump ease of use translate into higher overall satisfaction at 12 months, as reflected by the QoLSPP total score? As a secondary objective, do patient-reported outcomes differ between the Infla10 X and Infla10 AX models at 12 months?

## Methods

### Study design

This was a retrospective, single-center, single-surgeon, record-based cohort study. Pump type was not randomly assigned and was era-dependent; therefore, pump-related comparisons were considered exploratory and interpreted with attention to temporal confounding.

### Study population

A total of 299 patients were initially identified among men who underwent primary implantation of a Rigicon Infla10 X or Infla10 AX device between January 2021 and December 2024. Record-based 12-month outcome data were collected from routinely documented follow-up records beginning in May 2025. Only patients who had completed at least 12 months of postoperative follow-up at the time of data extraction were eligible for inclusion. Patients were included if they had complete 12-month record-based patient-reported outcome documentation, including IIEF-EF scores, QoLSPP total score, and NRS (1-10) ratings for inflation and deflation ease. During the study period, two different pump systems were used in routine practice, reflecting institutional adoption over time (earlier-generation pump and Pulse pump). Patients were excluded if they had a history of prior penile prosthesis implantation; underwent revision or salvage procedures; received a non-Rigicon device; had concomitant major reconstructive surgery (eg, urethroplasty or artificial urinary sphincter implantation); or had incomplete 12-month outcome documentation in the medical record. After applying these criteria, the final analytic cohort comprised 275 patients. All consecutive eligible cases meeting inclusion criteria with complete 12-month documentation were included in the final analysis.

### Ethics approval and consent

Ethical approval for the study was obtained from the Clinical Research Ethics Committee of Altınbaş University (approval number: 2024/42 IRB). Given the retrospective design and the use of fully anonymized record-based data, the Ethics Committee waived the requirement for informed consent.

### Data collection and clinical variables

Demographic data, including age, smoking status, and comorbidities such as DM and coronary artery disease, were extracted from the medical record. Device-related variables included IPP model (Rigicon Infla10 X vs Rigicon Infla10 AX) and pump type (earlier-generation Rigicon pump vs Pulse pump). In addition to conventional satisfaction outcomes, the study specifically evaluated patient-reported device usability parameters related to pump handling and functional ease of operation in routine clinical practice. Preoperative erectile function was assessed using the International Index of Erectile Function–Erectile Function domain (IIEF-EF). Operative time was recorded for each procedure and defined as the duration from skin incision to completion of skin closure, obtained from operative records and included as a perioperative variable for descriptive and comparative analyses.

### Pump systems

Medical record documentation indicated that two pump systems were used during the study period (January 2021-December 2024). The Pulse pump became commercially available in April 2024 and appeared in our clinical records thereafter. Cases were categorized by pump type as either the earlier-generation Rigicon pump or the Pulse pump. The Pulse pump appeared in the latter part of the study period and, once it was implemented in routine practice, subsequent implantations used the Pulse pump exclusively, with no further routine use of the earlier-generation pump. Because pump type was not randomly assigned and was strongly associated with calendar time, comparisons between pump groups were interpreted with attention to temporal confounding.

### International Index of Erectile Function

Erectile function was assessed using the Erectile Function domain of the IIEF-15 questionnaire (IIEF-EF), which comprises six items (questions 1, 2, 3, 4, 5, and 15). Each item is scored on a 5-point Likert scale, yielding a total score range of 6-30.^20^ The IIEF-EF was documented preoperatively and at the 12-month postoperative follow-up as part of routine clinical records. For Turkish-speaking patients, a previously validated Turkish version of the IIEF was used. The English version was administered to patients who were fluent in English.

### Quality of life and sexuality with Penile Prosthesis

Postoperative quality of life and sexual satisfaction were evaluated using the Quality of Life and Sexuality with Penile Prosthesis (QoLSPP) questionnaire.^21^ The QoLSPP assesses four domains (functional, personal, relational, and social) on a 0-5 scale, with higher scores indicating greater satisfaction. In accordance with subsequent clinical applications of the instrument, results were analyzed and reported as total QoLSPP scores, calculated as the mean of all items, yielding a total score range from 0 to 5. QoLSPP outcomes were documented at the 12-month postoperative visit as part of routine follow-up records and were extracted for analysis. The QoLSPP “ease of use” result reflects the relevant QoLSPP item/domain score (0-5 scale) and is distinct from the separate 1-10 NRS used for inflation and deflation ease described below. Because the QoLSPP instrument was originally developed and validated in Italian, and no formally validated Turkish version was available during the study period, the questionnaire was administered using routine clinical translation for Turkish-speaking patients. The absence of formal psychometric validation in Turkish represents a methodological limitation and results should be interpreted with appropriate caution.

### Pump inflation and deflation ease

Patients rated the ease of pump inflation and deflation using a NRS ranging from 1 (very difficult) to 10 (very easy). These assessments were performed during routine follow-up visits and were recorded by nursing staff as part of routine clinical documentation.

### Perioperative management

All patients received perioperative intravenous antibiotic prophylaxis according to institutional protocol prior to skin incision. No postoperative oral antibiotics were routinely prescribed. The prophylaxis regimen remained consistent throughout the study period, and deviations were made only for documented β-lactam allergy.

### Surgical technique

All procedures were performed by a single surgeon using a penoscrotal approach. The choice between Rigicon Infla10 X and Infla10 AX models was based on anatomical considerations and surgeon judgment, with patient preference incorporated when documented in the preoperative assessment. Reservoirs were routinely placed in the right retropubic space, and pumps were positioned in a midline dartos pouch. Surgical drains were not routinely used. Postoperatively, devices were typically left partially inflated, and a scrotal compression dressing (mummy wrap) was applied for approximately 24 hours per protocol.

### Statistical analysis

Continuous variables were assessed for normality using the Kolmogorov–Smirnov test. Normally distributed variables are presented as mean ± standard deviation and were compared using the independent-samples *t*-test. Non-normally distributed variables were analyzed using the Mann–Whitney U test. Categorical variables are presented as frequencies and percentages and were compared using the chi-square test or Fisher’s exact test where appropriate.

The primary endpoint was overall postoperative satisfaction at 12 months as assessed by the QoLSPP total score. Secondary endpoints included changes in erectile function (IIEF-EF), pump inflation and deflation ease measured on a 1-10 NRS, and perioperative complication rates.

Subgroup analyses were performed according to implant model (Infla10 X vs. Infla10 AX) and pump type (earlier-generation Rigicon pump vs. Pulse pump). Because pump type was strongly associated with calendar time and not randomly assigned, multivariable adjustment was not feasible due to collinearity; therefore, pump-related comparisons should be interpreted as exploratory and hypothesis-generating.

No adjustment for multiple comparisons was applied. Subgroup and pump-type analyses were considered exploratory in nature. A two-sided *P* value <.05 was considered statistically significant. All analyses were performed using IBM SPSS Statistics v26.0 (IBM Corp., Armonk, NY, USA).

All statistical tests were two-sided, and a *P*-value <.05 was considered statistically significant. No adjustment for multiple comparisons was applied. Subgroup and pump-type analyses were considered exploratory in nature.

## Results

### Baseline demographic and clinical characteristics

Baseline demographic and perioperative characteristics stratified by prosthesis model (Rigicon Infla10 X vs. Infla10 AX) are presented in Table 1A. No statistically significant differences were observed between implant groups across baseline erectile function, 12-month postoperative IIEF-EF, QoLSPP total score, or surgery duration (all *P* > .05). Comparative analyses stratified by pump type (earlier-generation Rigicon pump vs. Pulse pump) are presented in Table 1B.

**Table 1A:** Baseline and 12-month outcomes according to prosthesis model.

**Variable**	**Rigicon Infla10 X (*n* = 113)**	**Rigicon Infla10 AX (*n* = 162)**	***P*-value**
**Age (years)**	59.68 ± 7.45	59.67 ± 7.38	.997
**Preoperative IIEF-EF**	6.00 ± 1.54	6.16 ± 1.42	.365
**12-month postoperative IIEF-EF**	23.99 ± 1.91	24.12 ± 1.68	.534
**QoLSPP total score (sum, 0-45)**	37.8 ± 1.62	37.8 ± 1.62	37.8 ± 1.62
**Surgery duration (min), *n* (%)**			.195
**30-45**	77 (68.1)	108 (66.7)	
**45-60**	27 (23.9)	43 (26.5)	
**60-90**	9 (8.0)	11 (6.8)	

**Table 1B:** Baseline and 12-month outcomes according to pump type.

**Variable**	**Earlier-generation pump**	**Pulse pump**	***P*-value**
**Inflation ease (NRS 1-10)**	6.78 ± 1.44	7.61 ± 0.66	<.001
**Deflation ease (NRS 1-10)**	6.83 ± 0.91	7.69 ± 0.54	<.001
**12-month postoperative IIEF-EF**	23.63 ± 1.62	25.96 ± 1.03	<.001
**QoLSPP total score** **(sum, 0-45)**	37.6 ± 1.35	37.9 ± 1.53	.821

### Perioperative outcomes and complications

The overall complication rate in the cohort was 5.5%. Perioperative and early postoperative events included corporal crossover in 6 patients (2.1%), scrotal hematoma in 7 patients (2.4%), proximal corporal perforation in 2 patients (0.7%), and device infection in 1 patient (0.4%). No additional major adverse events were recorded during the 12-month follow-up period.

### Erectile function outcomes (IIEF-EF)

Changes in erectile function over time are presented in Table 1A and Table 1B. The mean preoperative IIEF-EF score was 6.09 ± 1.47, which increased significantly to 24.07 ± 1.78 at 12 months postoperatively, indicating a substantial improvement in erectile function. The narrow preoperative IIEF-EF score range (6-9) reflects the inclusion of patients with severe ED who were eligible for surgical intervention after failure of medical management.

### Postoperative quality of life outcomes (QoLSPP)

Postoperative patient-reported quality-of-life outcomes assessed using the QoLSPP questionnaire are summarized in Table 2. In accordance with subsequent clinical applications of the instrument, results are presented as total QoLSPP scores at 12 months.

**Table 2:** Postoperative QoLSPP domain scores at 12 months in the present cohort (*n* = 275).

**Domain**	**Item**	**Mean score (0-5)**
**Functional**	Prosthesis adequacy	4.6
	Ease of use	4.0
	Penile rigidity	4.6
**Personal**	Liveliness and wit	4.5
	Security	4.5
**Relational**	Well-being of the couple	4.2
	Partner satisfaction	4.0
**Social**	General well-being	4.3
	Feeling like others	4.2
**Overall**	QoLSPP total mean	4.2

The overall postoperative QoLSPP total score was 4.19 ± 0.16 (range: 3.7-4.6) on a 0-5 scale, with higher scores indicating greater satisfaction. Values above 4.0 are generally interpreted as reflecting high levels of postoperative satisfaction in previously published series. Patients in the Infla10 X group reported a mean QoLSPP score of 4.20 ± 0.18 (range: 3.8-4.6), while those in the Infla10 AX group had a mean score of 4.18 ± 0.16 (range: 3.7-4.5). This difference was not statistically significant (*P* = .436).

### Pump type–specific functional and satisfaction outcomes

Pump-related outcomes stratified by pump type are presented in Table 1B. Inflation and deflation ease scores, assessed using a 1-10 NRS, were significantly higher in the Pulse pump group compared with the earlier-generation pump group (inflation: 7.61 ± 0.66 vs. 6.78 ± 1.44; deflation: 7.69 ± 0.54 vs. 6.83 ± 0.91; both *P* < .001).

At 12 months, IIEF-EF scores were also significantly higher in the Pulse pump group compared with the earlier-generation pump group (25.96 ± 1.03 vs. 23.63 ± 1.62, *P* < .001). In contrast, postoperative QoLSPP total scores did not differ significantly between pump groups (4.21 ± 0.17 vs. 4.18 ± 0.15, *P* = .821). Overall, patient-reported satisfaction remained high across pump types at 12 months.

## Discussion

ED remains a prevalent and challenging condition, particularly in patients unresponsive to conservative therapies. IPPs offer a durable and effective solution in such cases, restoring functional erections through mechanical means.^19^ In our study, Rigicon Infla10 prostheses were associated with significant improvements in erectile function, as evidenced by an increase in IIEF-EF scores from preoperative to postoperative assessments. Postoperative satisfaction was also high, with QoLSPP total scores indicating favorable overall patient-reported outcomes at 12 months. These findings suggest that the Rigicon Infla10 X and Infla10 AX models not only restore sexual function but also contribute meaningfully to postoperative sexual quality of life. Importantly, no statistically significant difference was found between the two models in overall QoLSPP scores, which may indicate that the additional length-expanding feature of the Rigicon Infla10 AX model does not significantly alter perceived satisfaction at one-year follow-up. Our QoLSPP scores were contextually interpreted in relation to previously published data for other IPP models. Luna et al.^22^ and Di Pierro et al.^23^ reported postoperative QoLSPP outcomes with AMS 700 implants, while Colombo et al.^24^ evaluated satisfaction after ZSI 475 implantation. These comparisons, although informative, should be interpreted cautiously due to differences in device models, patient characteristics, and study methodologies.

The Pulse pump, a newer-generation pump system by Rigicon introduced during the later phase of this study, was associated with significantly higher ease-of-use scores for both inflation and deflation when compared to the earlier-generation pump. These findings suggest that pump-related handling characteristics may influence patient-perceived usability. Ease of inflation and deflation may contribute to overall patient satisfaction and usability perceptions. A recent prospective study by Cinar et al. reported that adoption of the Pulse pump was associated with higher ease-of-use ratings and improved functional outcomes.^19^

Despite these functional improvements, overall QoLSPP total scores remained consistently high across pump types. This apparent discrepancy between the numeric ease-of-use ratings and the QoLSPP results likely reflects differences in measurement focus. The 1-10 NRS captures task-specific mechanical handling of inflation and deflation, whereas the QoLSPP instrument integrates broader functional and psychosocial constructs. In a cohort with high overall satisfaction (mean QoLSPP >4.0 on a 0-5 scale), a potential ceiling effect may further limit the ability of the QoLSPP total score to detect incremental ergonomic improvements between pump designs.

While caution is necessary when comparing across different patient populations and methodologies, our findings align with the generally favorable satisfaction outcomes reported in the broader IPP literature. However, beyond overall satisfaction assessment alone, the present study additionally focused on patient-reported device usability and handling-related outcomes, which remain relatively underreported for the Rigicon Infla10 platform in routine clinical practice.

Nonetheless, this study has several limitations. First, its retrospective, single-center design may introduce selection bias and limits causal inference. In addition, several potentially relevant clinical variables were not consistently available (eg, prior pelvic surgery or radiation, presence of Peyronie’s disease, and history of prior penile prosthesis surgery), which restricted adjustment for confounding. Second, the 12-month follow-up period is insufficient to fully evaluate long-term mechanical reliability, device survival, and revision rates; extended follow-up has been shown to be important for assessing durability and late-onset complications in other IPP platforms.^25^ Third, although validated instruments were used, outcomes relied primarily on patient-reported measures (IIEF-EF and QoLSPP), which are inherently subjective and may be influenced by expectations, psychological factors, or recall bias. Fourth, the Pulse pump cohort represents a smaller and more recent subset of the study period; therefore, comparisons between pump types may be affected by performance bias related to temporal trends, surgeon learning-curve effects, and differential completeness of 12-month patient-reported follow-up. Fifth, the study was conducted in a high-volume center, which may limit generalizability to lower-volume settings. Finally, comparisons with previously published AMS and ZSI series^22–25^ represent historical rather than parallel controls; differences in patient characteristics, surgical technique, and follow-up protocols may confound cross-study comparisons and should be interpreted cautiously. Although a validated Turkish version of the IIEF was used, the QoLSPP questionnaire does not have a formally validated Turkish adaptation. The use of clinical translation in routine practice may introduce measurement variability and should be considered when interpreting patient-reported outcomes.

All implantations were performed by a single surgeon, supporting procedural consistency across the cohort. This consistency, combined with a high annual implant volume, may contribute to the low complication rate observed in our series, aligning with multicenter evidence that higher surgeon volume is associated with greater satisfaction and fewer complications.^26^

The newer Pulse pump was associated with higher patient-rated ease-of-use scores, although overall satisfaction remained high across pump types.Continued evaluation of long-term outcomes and multicenter prospective studies are necessary to confirm these findings and to further define the role of Rigicon IPPs in the evolving landscape of ED treatment.

Future studies may benefit from incorporating implant-specific satisfaction instruments such as the Satisfaction Survey for Inflatable Penile Implant (SSIPI). Compared with QoLSPP, which captures broader psychosocial and relational domains, SSIPI provides greater granularity regarding device-specific handling, mechanical function, and user interaction. The use of a more implant-focused instrument may allow detection of differences related to pump ergonomics, particularly when evaluating usability components such as inflation and deflation ease.

## Supplementary Material

Ethics_committee_approval_letter_qfag058

STROBE-checklist-FINAL-filled_qfag058

## Data Availability

Additional data are available from the corresponding author on reasonable request.
