# Supplementary material for: Efficacy, reliability, and patient satisfaction with Rigicon Infla10 X and Infla10 AX inflatable penile prostheses: a retrospective single-center analysis
Source: Sex Med. 2026 Jul 12;14(5):qfag058. doi: 10.1093/sexmed/qfag058 (PMC13356898; doi:10.1093/sexmed/qfag058)
Supplement: Ethics_committee_approval_letter_qfag058 [file ethics_committee_approval_letter_qfag058.pdf]

ALTINBAŞ ÜNİVERSİTESİ
SAĞLIK BİLİMLERİ BİLİMSEL ARAŞTIRMA ETİK KURULU

Sayı: 38 **Tarih: 10.01.2025**
Konu: Dr. Öğr. Üyesi Buğra Çetin

Sayın Dr. Öğr. Üyesi Buğra Çetin
Altınbaş Üniversitesi Tıp Fakültesi

İlgi: Altınbaş Üniversitesi Tıp Fakültesi Dekanlığının 01.11.2024 tarihli yazısı ile Altınbaş Üniversitesi Sağlık Bilimleri Bilimsel Araştırma Etik Kurulunun 2024/42 sayılı yazısı.

Sorumlu araştırıcılığını üstlendiğiniz **2024/42** dosya numaralı "**Rigicon Infla10 Penis İmplantı Sonrası Yaşam Kalitesi ve Cinselliğin Değerlendirilmesi: QoLSPP Anketinden Elde Edilen Görüşler**" başlıklı çalışma, kurulumuzun **10 Ocak 2025 tarih ve 1 sayılı toplantısında** görüşülerek etik yönden uygun bulunmuştur ve tutanaklar ekte sunulmuştur.

Bilgilerinize sunarım.

Prof. Dr. Mustafa Aydın BARLAS

Sağlık Bilimleri Bilimsel Araştırma

Etik Kurul Başkanı

E-imzalıdır

Eki: Altınbaş Üniversitesi Sağlık Bilimleri Bilimsel Araştırma Etik Kurulu Karar Formu

Bu belge, 5070 sayılı Elektronik İmza Kanununa göre Güvenli Elektronik İmza ile imzalanmıştır.
Evrak sorgulaması https://www.turkiye.gov.tr/altinbas-universitesi-ebys?eD=BSL3ST0J02&eS=79287 adresinden yapılabilir.

ALTINBAŞ ÜNİVERSİTESİ SAĞLIK BİLİMLERİ BİLİMSEL ARAŞTIRMA ETİK KURULU KARAR FORMU

BAŞVURU BİLGİLERİ					
ARAŞTIRMANIN AÇIK ADI	**Rigicon Infla10 Penis İmplantı Sonrası Yaşam Kalitesi ve Cinselliğin Değerlendirilmesi: QoLSPP Anketinden Elde Edilen Görüşler**				
ARAŞTIRMA PROTOKOL KODU	**2024/42**				
KOORDİNATÖR/SORUMLU ARAŞTIRMACI UNVANI/ADI/SOYADI	**Dr. Öğr. Üyesi Buğra Çetin**				
KOORDİNATÖR/SORUMLU ARAŞTIRMACININ UZMANLIK ALANI	**Üroloji**				
KOORDİNATÖR/SORUMLU ARAŞTIRMACININ BULUNDUĞU MERKEZ	**Altınbaş Üniversitesi Tıp Fakültesi**				
ARAŞTIRMANIN TÜRÜ	Anket	□			
	Bilimsel araştırma	■			
	Diğer ise belirtiniz:				
ARAŞTIRMAYA KATILAN MERKEZLER	TEK MERKEZ ■	ÇOK MERKEZLİ □	ULUSAL ■	ULUSLARARASI □	

DEĞERLENDİRİLEN BELGELER			
1	İNDEKS	■	DİĞER:
2	AKADEMİK KURUL / KURUM KARARI	■	
3	OLGU RAPOR FORMU	□	
4	BAŞVURU FORMU	■	
5	HASTA TAKİP FORMU ÖRNEĞİ	■	
6	BİLGİLENDİRİLMİŞ GÖNÜLLÜ OLUR FORMU ÖRNEĞİ	■	
7	ARAŞTIRMA BÜTÇESİ	■	
8	DESTEKLEYEN KURULUŞ	□	
9	LİTERATÜR ÖRNEĞİ	■	
10	TAAHHÜTNAME	■	
11	İZİN BELGELERİ	□	
12	ÖZGEÇMİŞLER	■	

KARAR BİLGİLERİ		
**Karar No: 1**		**Tarih: 09.01.2025**
		Yukarıda bilgileri verilen araştırma başvuru dosyası ile ilgili belgeler araştırmanın gerekçe, amaç, yaklaşım ve yöntemleri dikkate alınarak incelenmiş, gerçekleştirilmesinde **etik ve bilimsel sakınca bulunmadığına** toplantıya katılan **Sağlık Bilimleri Bilimsel Araştırma Etik Kurul** üye tam sayısının salt çoğunluğu ile karar verilmiştir.

ALTINBAŞ ÜNİVERSİTESİ SAĞLIK BİLİMLERİ BİLİMSEL ARAŞTIRMA ETİK KURULU

Unvanı/Adı/Soyadı	Uzmanlık Alanı	Kurumu	Cinsiyet		Araştırma ile ilişki *		Katılım **		İmza
Prof. Dr. Mustafa Aydın BARLAS (ETİK KURUL BAŞKANI)	Farmakoloji	**Altınbaş Üniversitesi**	E ■	K □	E □	H ■	E ■	H □	e-imzalıdır
Prof. Dr. Hatun Hanzade DOĞAN	Tıp Etiği	Altınbaş Üniversitesi	E □	K ■	E □	H ■	E ■	H □	e-imzalıdır
Prof. Dr. Nuray Yılmaz Altıntaş	Ağız Diş ve Çene Cerrahisi	Altınbaş Üniversitesi	E □	K ■	E □	H ■	E □	H ■	e-imzalıdır
Doç. Dr. Şükriye Karadayı	Adli Bilimler	Altınbaş Üniversitesi	E □	K ■	E □	H ■	E □	H ■	e-imzalıdır
Dr. Öğr. Üyesi Emir Ruşen	Nöroloji	Altınbaş Üniversitesi	E ■	K □	E □	H ■	E ■	H □	e-imzalıdır
Dr. Öğr. Üyesi Gaye HAFEZ	Farmakoloji	Altınbaş Üniversitesi	E □	K ■	E □	H ■	E ■	H □	e-imzalıdır

* :Araştırma ile İlişki
** :Toplantıda Bulunma

Bu belge, 5070 sayılı Elektronik İmza Kanununa göre Güvenli Elektronik İmza ile imzalanmıştır.
Evrak sorgulaması https://www.turkiye.gov.tr/altinbas-universitesi-ebys?eD=BSL3ST0J02&eS=79287 adresinden yapılabilir
