# Supplementary material for: Efficacy, reliability, and patient satisfaction with Rigicon Infla10 X and Infla10 AX inflatable penile prostheses: a retrospective single-center analysis
Source: Sex Med. 2026 Jul 12;14(5):qfag058. doi: 10.1093/sexmed/qfag058 (PMC13356898; doi:10.1093/sexmed/qfag058)
Supplement: STROBE-checklist-FINAL-filled_qfag058 [file strobe-checklist-final-filled_qfag058.docx]

STROBE Statement—checklist of items that should be included in reports of observational studies

|  | Item No. | Recommendation | Page  No. | Relevant text from manuscript |
| --- | --- | --- | --- | --- |
| **Title and abstract** | 1 | (*a*) Indicate the study’s design with a commonly used term in the title or the abstract | 1 | Title includes 'A Retrospective Single-Center Analysis'; Abstract provides a structured summary. |
|  |  | (*b*) Provide in the abstract an informative and balanced summary of what was done and what was found | 2 | Title includes 'A Retrospective Single-Center Analysis'; Abstract provides a structured summary. |
| Introduction | | | |  |
| Background/rationale | 2 | Explain the scientific background and rationale for the investigation being reported | 3 | Introduction outlines the clinical context and rationale for the study. |
| Objectives | 3 | State specific objectives, including any prespecified hypotheses | 4 | Clear objective stated: to evaluate efficacy, reliability, and patient satisfaction with Rigicon Infla10 X® and AX®. |
| Methods | | | |  |
| Study design | 4 | Present key elements of study design early in the paper | 4 | Described as a retrospective single-center study. |
| Setting | 5 | Describe the setting, locations, and relevant dates, including periods of recruitment, exposure, follow-up, and data collection | 4 | Setting: single center; timeframe: January 2021 to December 2024. |
| Participants | 6 | (*a*) *Cohort study*—Give the eligibility criteria, and the sources and methods of selection of participants. Describe methods of follow-up  *Case-control study*—Give the eligibility criteria, and the sources and methods of case ascertainment and control selection. Give the rationale for the choice of cases and controls  *Cross-sectional study*—Give the eligibility criteria, and the sources and methods of selection of participants | 4 | 299 patients underwent IPP placement; 24 excluded for non-participation; 275 included. |
|  |  | (*b*) *Cohort study*—For matched studies, give matching criteria and number of exposed and unexposed  *Case-control study*—For matched studies, give matching criteria and the number of controls per case | 4 | 299 patients underwent IPP placement; 24 excluded for non-participation; 275 included. |
| Variables | 7 | Clearly define all outcomes, exposures, predictors, potential confounders, and effect modifiers. Give diagnostic criteria, if applicable | 5,6 | Variables include IIEF-EF, QoLSPP, pump type, complications, and demographics. |
| Data sources/ measurement | 8 | For each variable of interest, give sources of data and details of methods of assessment (measurement). Describe comparability of assessment methods if there is more than one group | 5,6 | A validated instrument was used to assess erectile function (IIEF-EF), both preoperatively and at 12-month follow-up. Quality of life was evaluated using the QoLSPP questionnaire at the same time points. Ease of inflation and deflation was assessed with a numeric rating scale (NRS) by independent staff blinded to pump type. |
| Bias | 9 | Describe any efforts to address potential sources of bias | 4 | To minimize selection bias, all eligible patients who completed 12-month follow-up were included. |
| Study size | 10 | Explain how the study size was arrived at | 4 | All eligible patients treated during the study period were included. No formal sample size calculation was performed due to the retrospective design of the study. |

| Quantitative variables | 11 | Explain how quantitative variables were handled in the analyses. If applicable, describe which groupings were chosen and why | 6,7 | Continuous variables such as IIEF-EF scores, QoLSPP scores, and ease-of-use ratings were analyzed using means, standard deviations, and ranges. Comparisons between groups (e.g., standard pump vs Pulse® pump) were conducted using appropriate parametric or non-parametric tests depending on data distribution. No arbitrary cut-offs or categorizations were applied. |
| --- | --- | --- | --- | --- |
| Statistical methods | 12 | (*a*) Describe all statistical methods, including those used to control for confounding | 7 | Statistical analyses were performed using IBM SPSS Statistics v26.0. Continuous variables were compared using the independent-samples t test or Mann–Whitney U test according to distribution, and categorical variables using the chi-square test or Fisher’s exact test. No multivariable adjustment or multiple-comparison correction was applied; pump-type comparisons were considered exploratory because of collinearity with calendar time. |
|  |  | (*b*) Describe any methods used to examine subgroups and interactions | 6,7 | Subgroup analyses were performed by implant model (Infla10 X® vs. Infla10 AX®) and pump type (earlier-generation Rigicon pump vs. Pulse® pump). No interaction terms were tested. |
|  |  | (*c*) Explain how missing data were addressed | 5 | There were no missing data for primary or secondary outcome variables. |
|  |  | (*d*) *Cohort study*—If applicable, explain how loss to follow-up was addressed  *Case-control study*—If applicable, explain how matching of cases and controls was addressed  *Cross-sectional study*—If applicable, describe analytical methods taking account of sampling strategy | 4 | All patients included had completed 12 months of follow-up; no loss to follow-up occurred. |
|  |  | (*e*) Describe any sensitivity analyses | 5 | No sensitivity analyses were conducted. |
| Results | | | | |
| Participants | 13* | (a) Report numbers of individuals at each stage of study—eg numbers potentially eligible, examined for eligibility, confirmed eligible, included in the study, completing follow-up, and analysed | 4,5 | A total of 299 patients underwent primary IPP implantation with Rigicon Infla10® devices during the study period. Following application of the inclusion and exclusion criteria, 275 patients with complete 12-month documentation comprised the final analytic cohort. |
|  |  | (b) Give reasons for non-participation at each stage | 5 | Twenty-four patients were excluded from the final analysis due to incomplete 12-month documentation. |
|  |  | (c) Consider use of a flow diagram |  |  |
| Descriptive data | 14* | (a) Give characteristics of study participants (eg demographic, clinical, social) and information on exposures and potential confounders | 5 | Participant demographics including age, smoking status, diabetes mellitus (DM), coronary artery disease (CAD), anticoagulation status, and device type (Infla10 X® or AX®, standard or Pulse® pump) are presented in Table 1. |
|  |  | (b) Indicate number of participants with missing data for each variable of interest | 5 | There were no missing data for any of the primary or secondary variables of interest. |
|  |  | (c) *Cohort study*—Summarise follow-up time (eg, average and total amount) | 5 | All included patients completed the 12-month follow-up period, yielding a total of 275 patient-years of follow-up. |
| Outcome data | 15* | *Cohort study*—Report numbers of outcome events or summary measures over time | *6* | Erectile function improved significantly from a mean preoperative IIEF-EF score of 6.09 ± 3.21 to 24.07 ± 3.49 at 12 months (p < 0.001).  Overall QoLSPP scores were high (mean: 4.19 ± 0.29), indicating excellent patient satisfaction.  Patients using the Pulse® pump reported significantly higher ease-of-use scores than those using the standard pump (inflation: 7.61 vs 6.78; deflation: 7.69 vs 6.83; p < 0.001).  Postoperative complications occurred in 6.3% of patients, with scrotal hematoma (2.5%) and crossover (2.2%) being most common. |
|  |  | *Case-control study—*Report numbers in each exposure category, or summary measures of exposure |  |  |
|  |  | *Cross-sectional study—*Report numbers of outcome events or summary measures |  |  |
| Main results | 16 | (*a*) Give unadjusted estimates and, if applicable, confounder-adjusted estimates and their precision (eg, 95% confidence interval). Make clear which confounders were adjusted for and why they were included | 7 | Results were reported using unadjusted descriptive statistics including mean, standard deviation, range, and p-values. No multivariable analysis or confounder adjustment was performed because the aim was to describe outcomes and subgroup comparisons, not infer causality. |
|  |  | (*b*) Report category boundaries when continuous variables were categorized | 6 | No continuous variables were categorized in the analysis; all outcome measures (e.g., IIEF-EF, QoLSPP, NRS) were treated as continuous variables. |
|  |  | (*c*) If relevant, consider translating estimates of relative risk into absolute risk for a meaningful time period | 6 | Relative or absolute risks were not calculated, as the study design was descriptive and non-comparative with respect to risk outcomes. |

| Other analyses | 17 | Report other analyses done—eg analyses of subgroups and interactions, and sensitivity analyses 6 | Subgroup analyses were conducted to compare outcomes between prosthesis models (Infla10 X® vs Infla10 AX®) and between pump types (standard Rigicon pump vs Pulse® pump). No interaction analyses or sensitivity analyses were performed. |  | |
| --- | --- | --- | --- | --- | --- |
| Discussion | | | | |  |
| Key results | 18 | Summarise key results with reference to study objectives 8 | The main findings of this study indicate that implantation of Rigicon Infla10 X® and Infla10 AX® prostheses was associated with improvement in erectile function and generally favorable patient-reported outcomes at 12 months, based on IIEF-EF and QoLSPP results. In exploratory subgroup analyses, the newly introduced Pulse® pump was associated with higher ease-of-use scores compared with the earlier pump model. Overall, these results contribute to the evaluation of postoperative outcomes and patient-reported satisfaction following implantation of Rigicon inflatable penile prostheses. |  | |
| Limitations | 19 | Discuss limitations of the study, taking into account sources of potential bias or 10 imprecision. Discuss both direction and magnitude of any potential bias | Several limitations should be acknowledged. The retrospective single-center design may have introduced selection bias and limits causal inference. The 12-month follow-up duration does not allow robust assessment of long-term device durability, mechanical reliability, or revision outcomes. In addition, although erectile function was assessed using the validated IIEF-EF, the QoLSPP lacks a formally validated Turkish version, which may have affected the consistency of patient-reported outcome measurement. Pump-type comparisons should also be interpreted with caution because these analyses were exploratory and pump type was closely linked to calendar time. Finally, the study was conducted in a high-volume center, which may limit generalizability to lower-volume settings. |  | |
| Interpretation | 20 | Give a cautious overall interpretation of results considering objectives, limitations, multiplicity of analyses, results from similar studies, and other relevant evidence 9,10 | Overall, Rigicon Infla10 X® and Infla10 AX® prostheses were associated with improved erectile function and favorable patient-reported outcomes at 12 months, with no significant differences between implant models. Pump-type subgroup findings should be interpreted cautiously because analyses were exploratory, no adjustment for multiple comparisons was applied, and pump type was closely linked to calendar time. Interpretation is further limited by the retrospective single-center design, 12-month follow-up, and non-validated Turkish use of the QoLSPP. The findings are broadly consistent with previous reports of favorable short-term outcomes after IPP implantation, although cross-study comparisons remain limited. |  | |
| Generalisability | 21 | Discuss the generalisability (external validity) of the study results 9,10,11,12 | Generalisability may be limited by the single-center design, high-volume setting, and procedures being performed by a single experienced surgeon. Outcomes may differ in lower-volume or less specialized centers. In addition, the lack of a formally validated Turkish version of the QoLSPP may limit comparability across language and cultural settings. However, inclusion of both Rigicon Infla10 X® and AX® models and two pump types may enhance the relevance of the findings for other centers using similar devices. |  | |
| Other information | |  | | |  |
| Funding | 22 | Give the source of funding and the role of the funders for the present study and, if 1 applicable, for the original study on which the present article is based | The authors received no external funding for this study. The funding source had no role in study design, data collection, analysis, interpretation, or manuscript preparation. |  | |

*Give information separately for cases and controls in case-control studies and, if applicable, for exposed and unexposed groups in cohort and cross-sectional studies.

**Note:** An Explanation and Elaboration article discusses each checklist item and gives methodological background and published examples of transparent reporting. The STROBE checklist is best used in conjunction with this article (freely available on the Web sites of PLoS Medicine at http://www.plosmedicine.org/, Annals of Internal Medicine at http://www.annals.org/, and Epidemiology at http://www.epidem.com/). Information on the STROBE Initiative is available at www.strobe-statement.org.
